# Supplementary material for: Blood Glucose Prediction from Nutrition Analytics in Type 1 Diabetes: A Review
Source: Nutrients. 2024 Jul 10;16(14):2214. doi: 10.3390/nu16142214 (PMC11280346; doi:10.3390/nu16142214)
Supplement: Supplementary file 1 [file nutrients-16-02214-s001.zip › nutrients-3085381-supplementary.pdf]

Table S1: Summary of Physiological Blood Glucose Prediction Model Approaches

Author	Model Type	Additional Aspects	Sub-Systems	Prediction Horizon	Subjects used		Inputs Used					Accuracy
					Real Data	Simulated Data	CGM / BG	Simple carbohydrate	Mixed Meals	Insulin	Extra inputs	
Bergman, 1979 [50]	Glucose disappearance and insulin sensitivity	Three insulin-independent and four insulin-dependent models	Hepatic glucose balance and peripheral tissue uptake				x	x		x		“Good precision”
Lehmann, 1992 [51]	First order Euler integration	Glucose–insulin interaction, insulin sensitivity	Hepatic and peripheral glucose handling.	24 hr	1		x	x		x	Gastric emptying rate	1 mmol/L
Andreassen et al., 1994 [52]	Carbohydrate and insulin absorption models	Renal loss of glucose, insulin-independent and -dependent glucose utilization		24 hr	12		X	x		x		Average mean prediction error over day 2 for all patients 3.3mmol/L
Höfig et al.,1996 [53]	Extended mathematical models: 1. Exogenous glucose influx 2. Hepatic glucose production		Gastric emptying and small intestine absorption using Michaelis–Menten kinetics		11 Non-DM; 5 T1DM		X	x		x		
Arleth et al., 2000 [44]	Two-compartment carbohydrate absorption	DIAS	Gastric emptying rate. Factors affecting carbohydrate breakdown				X		Complex and GI ranking			
Dalla Man, 2007 [54]	Glucose–insulin control system		Glucose and insulin sub-systems		204		X		x			
Islam et al.,2007 [55]	Minimal and physiological models		Hepatic glucose balance. Glucose appearance rate				x	x		x		
Calm, 2011 [19]	Modal Interval Analysis (MIA)		Insulin absorption, carbohydrate digestion, and absorption insulin PK/PD	6 hr		1	x	x		x		

			glucose metabolism									
Elleri, 2013 [46]	Bayes model and two-compartment glucose kinetic model	Stable-label tracer dilution methodology	Stochastic modelling of glucose kinetics		8		x	Low GI v High GI		x		
Balakrishnan, 2014 [25]	Extended Bergman's minimal model absorption dynamics [48]	Roy and Parker exercise model	Meal absorption dynamics, insulin absorption kinetics, and exercise		34		x	x		x	Exercise	R^2 fitness: > 0.7 overall, 0.8–0.96 (82% pts). R^2 validation 0.75 –0.95 (88% pts)
Bock, 2015 [56]	Therapy parameter–based model (TPM)		Bergman minimal model [48] and Hovorka, Prud'homme model [19,51]	15–165 minutes	12	10	Finger pricks		x	x	Exercise	MAD: TPM 6.91 LMM 4.94 MM 5.26
Magdelaine et al., 2015 [57]	Glucose–Insulin and digestion models		Glucose dynamics, insulin dynamics, digestion dynamics, insulin sensitivity		Four hospitalized patients and one outpatient		x	x		x		SD +/- 20mg/dL per patient
Contreras, 2017 [28]	Hovorka model [58]	Grammatical evolution	Glucose, insulin, glucagon dynamics	6 hour (four segments)		100 UVA/ Padova T1D Simulator	x	x		x		98.31% EGA zones A and B
Liu et al, 2018 [59]	Discretized version of composite model; Forward Euler's configuration		Minimal model [48] and insulin/carbohydrate absorption model [51]	30/60/90/120 minutes	10	10 (UVa-Padova)	x		x (amount and absorption type)	x (bolus)	Exercise	Simulated: 26.68+/-3.58mg/dL (fasting); 23.89+/-3.32 (meal/exercise). Real: 37.02+/-5.14mg/dL (fasting); 35.96+/-4.65mg/dL (meal/exercise)
Rozendaal, 2018 [60]	Physiology-based dynamic modeling (PBDM) [60]	Glucose–insulin dynamics, carbohydrate digestion	Insulin–glucose kinetics			53 PPR datasets	X PPG			X	Gastric emptying (two terms: simple carbohydrate and	"Performs well for a variety of common foods and mixed meals"

											complex carbohydrate)	
Gyuk et al.,2019 [61]	Arleth model with personalized mathematical model [43,62]		Glucose and insulin absorption (differential equations)	30–60 minutes	26 T1 and insulin dep T2		x		x	x		RMSE (mmol/L): 3.00 (1 hr); 6.79(2 hr); 9.10(4 hr); 9.44(6 hr) | EGA (A+B acceptable) 89% (1 hr); 67% (2 hr); 57% (4 hr); 57% (^ hr)
Liu et al., 2019 [62]	Bergman [48] and Hovorka models [51]		Glucose–insulin dynamics	30/60/90/120 minutes	10 pts	UVa/Padova	x	x		x	Optional input of meal absorption models	PMma RMSE (mg/dL) 17.67 +/- 2.12 (30m); 30.36+/-3.88 (60m); 38.14+/-5.25; 40.46+/-6.06 (120m)
Karim et al., 2020 [7]	Arleth model [43]	FNN quasi-newton method. FNN-ABS (absorption), FNN-NUT (raw nutrients quantity), and FNN-NUT-GI	Stomach and Intestinal compartments, glucose absorption	60 minutes	5		x		x	x	Gastric emptying	1.12 mmol/L
Munoz-Organero, 2020 [16]	Modified Lehmann and Deutsch model	Recurrent neural network using long short-term memory		30–60 minutes	9 D1NAMO dataset	40 AIDA simulator	x	x		Fast and slow acting	BG history	RSME (30 minutes): <0.28 mmol/L (simulated), < 0.55 mmol/l (real). Clinically acceptable 90% (60 minutes), 99% (30 minutes)

Table S2: Summary of Data-Driven Blood Glucose Prediction Model Approaches

Author	Model Type	Additional Aspects	Prediction Horizon	Subjects Used		Inputs Used					Accuracy
				Real Data	Simulated Data	CGM / BG	Simple Carbohydrate	Mixed Meals	Insulin	Extra Inputs	
Tresp and Briegel, 1997 [63]	Non-linear recurrent neural predictive model	Forward–backward Kalman filter				x	x (times and amounts of Food—fast/int/ slow CHO)		x (basal and	Times and duration of exercise	
Sandham et al., 1998 [64]	Recurrent artificial neural network			2		x	x		x	Exercise, additional (stress/ surgery/ illness/ pregnancy)	
Bremer and Gough, 1999 [15]	Autocorrelation function (ACF)		10/20/30 minutes	22 datasets							RMSE 0.2mmol/L (10 minutes)
Ståhl, Johansson, and Renard, 2010 [65]	Black-box finite impulse response (FIR)		20 and 60 minutes	18		x	x		x		CGA 94.2% Zone A
Cescon and Renard, 2011 [66]	Data-driven multi-step subspace-based patient-specific predictor models		30 minutes			x	x		x	Exercise (heart rate)	
Pappada et al, 2011 [67]	Feed-forward neural network model		75 minutes	10		x		x	x	Lifestyle and emotional factors	RSME 43.9mg/dL +/- 6.5 and MAD 22.1; CEGA—zone A 62.3%, zone B 30.0%
Pappada, Cameron, and	Neural network model		50/75/ 100/120/	18		x		x (nutrition	x	Sleep–wake cycles,	

Rosman, 2008 [68]			180 minutes					al intake)		exercise	

Robertson et al., 2011 [69]	Elman recurrent artificial neural network	AIDA, Levenberg–Marquardt algorithm	60 minutes			x		x	x		RMSE (5 day)0.15+/-0.4SD mmol/L with error(max) 0.24mmol/L
Daskalaki et al., 2012 [70]	AR vs ARX vs ANN models	Tikhonov regularization	30/ 45 minutes		30–10 adults, 10 adolescents , 10 children	x	x (meals with set CHO ranges at set times)		x (basal / bolus regime based on ICR)		RSME—AR: 14.0–21.6mg/dL (30m), 23.2–35.9mg/dL (45min) / ARX: 13.3–183.8mg/dl (30m), 22.8–29.4mg/dL (45m) / ANN: 2.8–6.3mg/dL (30 and 45 minutes)
Pappada and Cameron, 2012 [71]	Train developed time-lagged feed forward neural network model (TLFFNNM)	Proportional integral derivative (PID)	75 minutes		10 (2 T1DM)	x	x		x	Sleep–wake cycles and sleep quality, exercise, emotional states	CEGA: 92.3% zones A and B (clinically acceptable), 62.3% zone A
van Heusden et al., 2012 [72]	Zone model predictive control (ZMPC)					x (AP)			x (AP)		
Steil, 2013 [73]	Proportional integrative derivative (PID)										
Toffanin et al., 2013 [74]	Multiple-model predictor (MMP)		60 minutes	18		x	x			Meal absorption / day period	

Cameron et al., 2014 [75]	Multiple model probabilistic predictive controller (MMPPC)	Kalman filter	5 hours	4 pts/6 pts		x		x unannounced		Exercise (walking <20 minutes between blood draws)	

Del Favero et al., 2014 [76]	Model predictive control (MPC)			6		x	x		x		Clinically acceptable: 94.5% (open loop); 88.3% (closed loop). TIR: 94.83% vs 68.2% (MPC vs open loop); hypo-events: 1.25 vs 11.9% (MPC vs open loop)
Efendic et al., 2014 [77]	Gaussian mixture models	Biometric and biological systems	10/20/ 30 minutes	12		x	x		x	Days 2 and 5 fastabsorbing foods, 15% increased and 15 minutes delayed insulin bolus	96.53% correct prediction rate
Kirchsteiger et al., 2014 [78]	CT identification algorithm	CHO and insulin sensitivity factor		28		x	x			Protein and fat intake	
Oveido et al., 2014 [79]	Personalized hybrid models using grammatical evolutions and physiological models	Gaussian distributions	120 minutes		100 in silico	x	x		x		RSME 1am–7am 11.8; 7am–1pm 22.09; 1pm–7pm 21.43; 7pm–1am 29mg/dL
Plis et al.,2014 [80]	Support vector regression	Physiological features; Gaussian kernel	30/60 minutes			x	x (meal absorption dynamics—CHO consumption; CHO digestion)		x	Hypo prediction	SVR : 22.6 (30 minutes); 35.8 (60 minutes)/ ARIMA : 24.9 (30 minutes); 39.6 (60 minutes)

Ståhl, Johansson, and Renard, 2014 [81]	Sliding window Bayesian model average predictor	Gaussian distribution	40 minutes		12 pt datasets	x		x (announced 30 minutes ahead)	x		RSME 1.03
Wang, 2014 [82]	Time-varying state space model		30 minutes	1	30	x	x		x		
Georga et al., 2015 [18]	Non-linear: on-line sequential extreme learning machine (OS-ELM) and online sequential extreme learning machine kernels (KOS-ELM)	10-fold cross validation	30 minutes	15		x	x		x	Physical activity	ELM: case 1 (15.7 +/- 3.0); case 2 (11.8 +/- 2.2); case 3 (9.3 +/- 1.9); K-ELM: case 1 (15.3 +/- 3.0); case 2 (8.6 +/- 2.2) ; case 3 (96.1 +/- 1.6); OS-ELM: case 1 (15.8 +/- 2.9); case 2 (13.9 +/- 2.3) ; case 3 (13.3 +/- 12.3); KOS-ELM: case 1 (16.6 +/- 2.69); case 2 (10.9 +/- 2.7); case 3 (8.5 +/- 2.7)

Georga et al., 2015 [83]	Random forest and RReliefF	Support vector regression or Gaussian process	30/60 minutes	15		x		x (type of food, serving size, and time)	x (type, dosage, and time)		Average RMSE (30 minutes): SVR/RF 21.4 +/- 7.5; SVR/RReliefF 24.9 +/- 5.9; GP/RF 22.9 +/- 9.7, GP/RRrelief 25.36 +/- 7.4 Average RMSE (60 minutes): SVR/RF 26.5 +/- 9.5, SVR/RReliefF 23.5 +/- 7.6; GP/RF 24.6 +/- 12.6 GP/RRelief26.4 +/- 11.6
Bazaev and Pozhar, 2017 [84]	Sigma model	Bennett–Gourley model for NN training		52 (tracks)		x		Food intake	x		RMSE: 15.7mg/dL, algorithm corrects 97.5% of errors
Hidalgo et al., 2017 [85]	1) A variant of grammatical evolution that used optimized grammar. 2) A variant of tree-based genetic programming that used a three-compartment model for CHO and insulin	Genetic programming and grammatical evolution compared to KNN RF and two base line predictors	30/ 60/ 90/ 120 minutes	10				Food intake	x		90% zones A and B (Clarke Error Grid)

Jankovic et al., 2016 [86]	Deep prediction model (DPM)	ARX, ANN used for prediction layer and ELM (correction layer)	15/30/45 minutes	6		x	x		x	Physical activity	Model results (15 minutes): ARX + ELM: 9.6 (2.15) ARX without ELM: 9.9 (1.59) cARX + ELM: 0.97 (0.01) cARX without ELM: 12.5 (1.63) ARXK + ELM: 9.4 (1.78) ARXK without ELM: 10.0 (1.83) ANN + ELM: 8.5 (1.49) ANN without ELM: 9.6 (1.85)<br><br>Model results (30 minutes): ARX + ELM: 19.9 (4.39) ARX without ELM: 18.9 (3.61) cARX + ELM: 19.9 (4.39) cARX without ELM: 22.5 (4.19) ARXK + ELM: 20.6 (5.82) ARXK without ELM: 19.6 (4.31)

											ANN + ELM: 17.8 (4.35)<br>ANN without ELM: 17.8 (4.36)<br><br>Model results (45 minutes):<br>ARX + ELM: 22.4 (4.69)<br>ARX without ELM: 26.5 (5.72)<br>cARX + ELM: 22.4 (4.69)<br>cARX without ELM: 29.8 (6.76)<br>ARXK + ELM: 23.1 (5.82)<br>ARXK without ELM: 27.5 (6.89)<br>ANN + ELM: 24.4 (6.11)<br>ANN without ELM: 24.5 (6.34)
Zecchin et al., 2016 [87]	Jump neural network		30 minutes	10		x		x	x	Physical activity, stress	RSME 17.6mg/dl JNN; RSME 22.9mg/dl Ref NN
Reiter, Reiterer anddel Re, 2017 [88]	Bayesian network	Markov chain model		37		x	x		x		

Wang et al.,2017 [89]	L-MPC			10		x				Exercise day/alcohol intake day	
Acedo et al., 2018 [90]	Mathematical model	Swarm hybrid				x	x				
Buckingham et al., 2018 [91]	Model predictive control			12		x	x / fat	High-fat meal (>30% TEI	x	Over-estimated bolus/extended bolus	76.1% +/- 8% TIR
Li et al., 2018	Convolutional recurrent neural network	Time series prediction using LSTM and a signalconverter	30/60 minutes	10 real cases	10 (UVa/Padova)	x	x		x (bolus)		30 minutes: 9.38 +/- 0.71mg/dL (simulated) 21.07+/- 2.35mg/dL (real) 60 minutes: 18.87+/- 2.25mg/dL (simulated) 33.27+/- 4.79mg/dL (real)
Vahedi et al., 2018 [92]	Random rorest regressor and MLP (neural networks regressor)		30 minutes								

Akbari and Chunara, 2019 [93]	Multi-signal Gaussain process					x	x	x	x	Sleep, work, exercise, heart rate, galvanic skin temp, skin temp, air temp, and step count	
Borle, Ryan,and Greiner, 2019 [94]	K-nearest neighbors, supportvector regression, ANN, wavelet neural network, ridge regression, random forest regression, Gaussian process regression	“Kok” features, 10-fold cross validation		47		x	x		x	Physical activity (less than normal/normal/active/very active)	MAE: 2.91mmol/l; 7.1% relative improvement on baseline
Griva, Martínez, and Basualdo, 2019 [95]	Average long-term prediction model (ALTPM)	Kalman filter vs Wiener model	60 minutes	2 pts		x	x		x		All data at 60 minutes—RMSE (Wiener model) 24mg/dL and 45.4 mg/dL; 37.6mg/dL and 48.9mg/dL (Kalman filter)
Georga, Príncipe, and Fotiadis, 2019 [96]	Non-linear, recursive, multivariant prediction model	Kernel Hilbert space (quantized kernel least mean square — QKLMS-FB; kernel recursive least squares—KRLS-ALD)	15/60 minutes	15		x	x		x	physical activity	RSME of QKLMS-FM 13.1mg/dL (15 minutes); 37.7mg/dL (60 minutes) KRLS-ALD 10l.5mg/dL (15 minutes),

											31.8mg/dL (60 minutes)
Litinskaia et al., 2019 [97]	Sigma model-based algorithm			DirecNet Database		x	Food intake (un-announced)		x	Physical activity	
Padmaprith a, 2019 [98]	Recurrent neural network–long short-term memory	Adam optimizer		T2DM pop							RMSE 18.79mg/dL
Rodríguez-Rodríguez et al., 2019 [26]	Sequential input selection algorithm (SISAL)		30 minutes	28 pts		x		x	x	Exercise, heart rate, sleep schedule, circadian rhythms	RMSE 17.83mg/dL

Saiti et al., 2019 [99]	Support vector regression and an extreme learning machine	Autoregressive with exogenous inputs (ARX)	30/45/60 minutes			x		x	x		RMSE training: ARX: 23.24 (30m), 29.54 (45m), 35.13(60m) SVR: 22.34(30m), 28.64 (45m), 34.3 (60m), ELM: 23.96 (30m), 32.07 (45m), 35.49 (60m), Comb: 20.18 (30m), 26.48 (45m), 33.33(60m)<br><br>RMSE testing:

											ARX: 21.62 (30m), 27.02 (45m), 29.72(60m) SVR: 22.58(30m), 26.48 (45m), 31.17 (60m), ELM: 21.08 (30m), 27.20 (45m), 30.63 (60m); Comb: 20.36 (30m), 25.58 (45m), 26.48 (60m)
Alquah, Younes, and Aldudah, 2020 [100]	Neural network	One hidden layer NN; two hidden layer NN; Kalman filter		149					x	Gender, age, BMI, disease history, total daily insulin dose, diabetes type, smoking factor, genetic factor, creatine clearance, and accumulative glucose	RSME: 2.3265mg/dL
Amar et al.,2020 [101]	Artificial neural network	Gradually connected network (3)	30/60 minutes		30						

Balasooriya and Nanayakkara, 2020 [102]	Long short-term memory		30 minutes			x	Food (CHO, prot, fat)		x	Physical activity (light, sedentary, moderate, high); meal absorption;	79.97% accuracy; RMSE 22.2mg/dL

										drug absorption	
Camerlingo et al., 2020 [103]	Support vector machine (SVM) classifier, univariant probability density parametric. Models; mathematical models	Radial basis function (RBF) kernel		32			x (meal timing)		x	Age, weight, last meal's CHO, mealtime, CHO sum (1 hr, 4 hr,6 hr), Mean CGM (1 hr, 4 hr, 6 hr), Initial CGM, CGM rate (1 hr), observation time, distinct hypo treatments	AUROC equal to 0.754
Contador et al., 2020 [104]	Multi-objective grammatical evolution based on NSGA-II algorithm					x	x		x		
Hidalgo et al., 2020 [105]	Markov chains; grammatical evolution	Metropolis–Hastings algorithm; symbolic aggregate approximation (SAX); sampling importance resampling (SIR)		5		x	x		x	Active IOB	
Kriventsov et al., 2020 [106]	Support vector machines for regression	Decision tree	15/30/45/60 minutes			x	x		x	Heart rate andphysical activity	

Munoz-Organero, 2020 [16]	Recurrent neural network–long short-term memory		30/60 minutes	9 pts - D1NAMO dataset	40 - AIDA diabetes simulator	x	x		x	Insulin absorption and utilization (dependent and independent), renal clearance, and endogenous production	RMSE (mg/dL) 10 memory cells 70% training and 30% validation—6.42(30m) and 11.35 (60m)
Pavan et al., 2020 [107]	Shallow neural network; ensemble trees		30/60 minutes	6 - OhioT1DM dataset		x	x		x	Work and sleep routine	
Saiti et al.,2020 [99]	ARX and SVR models	Linear, bagging, and boost meta regressor	30/45/60 minutes	2 in hospital pts; 4 D1NAMO		x	x		x		RMSE (mg/dL) pt 1 with BAGGING metaR 14.76+/-0.25 (30m); 22.32+/-0.41 (45m); 28.98+/-0.56 (60m)
Song et al.,2020 [108]	Learning type model predictive control			29		x	x		x	Physical activity, alcohol intake	65.2% TIR
Sun et al.,2020 [109]	Latent variable (LV)-based Model		30/60 minutes	6 - OhioT1DM dataset		x	Meal		IOB, basal, bolus	Exercise, work, illness	RMSE (mg/dL) 19.37+/-2.87 (30m); 32.50+/-5.35 (60m)
Zhu et al.,2020 [110]	Generative sdversarial network (GAN)	Recurrent neural network with convolutional neural network layers	30/60 minutes	12		x	x		x	Physical activity, sleep, work, stress	RMSE 18.34 (30 minutes); 32.21 (60 minutes)

Alvarado et al., 2021 [111]	Takagi–Sugen–kang fuzzy rule-based system (TSK-FRBS)	What-if and agnostic scenarios	30/60/90/120 minutes	10		x	x		x		RMSE (mg/dL) 11.38 (training); 29.44 (testing)
Beauchamp et al., 2021 [112]	Two long short-term memory chain	Global average and ToD average	30/45/ 60/75/ 90 minutes	12 - OhioT1DM dataset		x	x		x	Physiological and lifestyle factors	N-BEATS.mean with pre-processed meals RMSE 8.84 (training); 6.16 (validation; N-BEATS.best RMSE 1.22 (with pre-processed meals)
Bhargav et al., 2021 [113]	MLP, LSTM, VAR, ensemble				40	x	x		x	Kidney functioning, liver insulin sensitivity, lower and upper glucose limit	RSME mmol/l test dataset MLP: 6.44/ LSTM: 5.67/ VAR: 7.16/ ensemble: 4.59
Cui et al.,2021 [114]	Recurrent self attention-based deep network	Fully connected feed-forward network with ReLU activation (in hidden dimension)	30/60 minutes	12 - OhioT1DM dataset		x	x		x		RMSE 17.82mg/dL (30m); 28.54mg/dL (60m)
De Bois et al., 2021 [115]	Fully convolutional neural networks (FCN) vs support vector regression			6 - OhioT1DM dataset; 6 T2DM (IDIAB)	10 T1DM dataset (simulated)	x	x		x		IDIAB RMSE 20.32+/-6.02 (SVR); OhioT1DM RMSE 20.10+/-2.34 (SVR)

De Falco et al., 2021 [116]	Grammatical evolution		30 minutes	12 - OhioT1DM dataset		x	x		x		MAE (average) 13.48mg/dL
Rabby et al., 2021 [117]	Stacked long short-term memory (LSTM)-based deep recurrent neural network (RNN)	Kalman smoothing technique (correction of inaccurate BG readings) and feature extraction module (meal, insulin, step count)	30/60 minutes	6 - OhioT1DM dataset		x	x		x	Sleep quality, illness level, stress levels, hypo-events, work intensity, exercise duration, heart rate, skin temp, acceleration	Mean RMSE (mg/dL) smoothed CGM 5.89 (30m), 17.24 (60m); Single LSTM 18.96 (30m), 30.88 (60m); Stacked LSTM 18.57 (30m), 30.32 (60m)
Sahin and Aydin, 2021	Artificial neural network	IOB, glucose absorption rate and activity on board models	30/60 minutes	6 - OhioT1DM dataset		x	x		x	Physical activity (steps)	RMSE (mg/dL): Primary model - 18.81 (30m), 30.89 (60m)
Shahid, Hussain, and Khan, 2021 [118]	Convolutional network (CNN) with modified recurrent network (RNN) using GRU (CGRU)		15/30/60 minutes			x	x		x	HBGI and IBGI (as optional inputs)	RMSE (mg/dL) — 4.84+/1.83 (15m); 6.04+/-1.84 (30m); 8.12+/-1.46 (60m)
Wang, 2021 [119]	Comparison of machine learning algorithms	Support vector machines vs random forests vs linear regression vs K-nearest neighbors regression (KNN) vs XGBoosted trees			10 Uva/Padova	x	x		x		LSTM RSME 7.55+/-0.19

Zaidi et al.,2021 [120]	Temporal convolution neural networks (TCNs)		30 minutes	97		x	x		x		RMSE (mg/dL) 16.77 +/- 4.587
Zhang, Flores, and Tran, 2021 [121]	Neural network architectures vs a reservoir computing model vs novel linear regression		30/60 minutes	12 - OhioT1DM dataset		x	x		x	Sleep quality, illness level, stress levels, hypo-events, work intensity, exercise duration, heart rate, skin temp, acceleration	RMSE (mg/dL) 30 minutes: MLR 14.16 (train), 18.39 (test); BRC 10.82 (test), 24.49 (test); DCNN 21.41 (train), 29.96 (test); Seq-to-seq LSTM 10.02 (train), 17.52 (test) 60 minutes: MLR 221.39 (train), 24.58 (test); BRC 13.95 (test), 28.79 (test); DCNN 35.84 (train), 38.97 (test); Seq-to-seq LSTM 18.22 (train), 26.44 (test)

Aashima et al., 2022 [122]	Combined ML models (LR, MLP, KNN, SVR, DCT, and EXT) with ensemble models (RF, EXT, bagging,Aboost, Gboost)	DCT expert used for predictions in: DCT-ABoost, DCT-EXT, DCT-Bagging, DCT-RF, DCT-GBoost	24 hours (15-minute intervals)		40 - AIDA simulator	x	x		x	Kidney functioning, liver insulin sensitivity, lower and upper glucose limit; pts weight; timestamp	Test/Train RMSE: Individual models: DCT: 0.202 / 2.027 EXT: 0.199 / 2.207 KNN: 0.667 / 2.374 MLP: 2.050 / 2.507 SVR: 2.160 / 2.642 LR: 2.908 / 2.777 TWO-STAGE models DCT-Aboost: 0.211 / 2.204 DCT-EXT: 0.199 / 2.207 DCT-Bagging: 0.200 / 2.208 DCT-RF: 0.199 / 2.208 DCT-GBoost: 0.203 / 2.208
Daniels, Herrero, and Georgiou, 2022 [123]	End-to-end deep multitask learning approach (neural networks)	Single-task learning, transfer learning, multi-task learning, (glycemic variability)	30/45/60/90/120 minutes	12 - OhioT1DM dataset		x	x		x	Sleep quality, illness and stress levels, hypo-events, work intensity, exercise duration, heart rate, galvanic skin response (GSR), skin	RMSE (mg/dL): 18.8+/-2.3 (30m); 25.3+/-2.9 (45m); 31.8+/-3.9 (60m); 41.2+/-4.5 (90m); 47.2+/-4.6 (120m)

										temp, air temp, step count	CEG: 93% clinically acceptable
Simone et al., 2022	ARX/ARMAX/ ARIMAX/BJ	PopOrd/CV/BIC/AIC	30/60/ 90/120 minutes	11		x	x		x	Insulin-to-carb ratio and insulin sensitivity factor	Best prediction of those tested: non-parametric approaches 29.8mg/dL and median COD 57.4% (60m)
Yang et al.,2022 [124]	Autonomous channel deep learning framework (asynchronous temporal sequences)	Multi-lag structure embedded in autonomous channel network	30/60 minutes	12 - OhioT1DM dataset		x	x		x	Sleep quality, illness and stress levels, hypo-events, work intensity, exercise duration, heart rate, galvanic skin response (GSR), skin temp, air temp, step count	RMSE (mg/dL) 18.93+/-2.16 (30m); 31.79+/-3.43 (60m)
Mordvanyuk, Torrent-Fontbona, and López [125]	K-nearest neighbor (KNN)	Sequences of meals where each sequence contains all the ordered meals of a time window of d days			11	x	x		x		Overall performance TPRs up to 0.88; 83% accuray; FNR and FPR significantly lower, from 0.25

											to 0.11

Jaloli and Cescon, 2023 [126]	CNN-LSTM based deep neural etwork	Windowed samples of past data with length three times the PH (3× PH), which was the optimal length of input for all PHs, are input to a stack of 1D convolutional and pooling layers, followed by an LSTM block containing two layers of LSTM, each containing 100 LSTM units, followed by two fully connected layers	30/60/ 90 minutes	Replace-BG and DIAdvisor datasets	Replace-BG dataset: 168<br><br>DIAdvisor data set: 59	X	X		X		90-minute PH: 17.30 ± 2.07 and 18.23 ± 2.97 mg/dL
Sun, Rashid, Askari, and Cinar, 2023 [127]	MPC (AP system)	Glucose–insulin metabolism dynamic; unscented Kalman filter				X					
Cui, Nolan, Daskalaki, and Suominen, 2023 [128]	LSTM backbone with hidden dimension D followed by a rectified linear unit (ReLU)	Hyper and hypo prediction<br><br>Matthew's co-efficient	30 minutes	OhioT1DM dataset	12	X	X		X		RMSE 18.23 ± 0.35 (hyperglycemia)<br>13.25 ± 0.17 (hypo)

Langarica, Rodrigues-Fernandez, Doyle, and Núñez, 2023 [129]	Input and state recurrent Kalman network (ISRKN)		30/60 minutes	UVA/ Padova simulator	10 virtual patients<br><br>6 Ambulatory individuals	X		X	X		RMSE in silico —9.38mg/dL (30minutes), 18.87mg/dL (60 minutes).<br>Real—8.88mg/dL (30 minutes), 19.90mg/dL (60 minutes)
Langarica, de la Vega, Cariman, Miranda, Andrade, Núñez, and Rodriguez-Fernandez, 2024 [130]	LSTM<br><br>Encoder–decoder (Enc-Dec)<br><br>Bidirectional encoder–decoder (Bi Enc-Dec)<br><br>Encoder–decoder with double attention (Enc-Dec DAttn)		30 minutes	UCHTT1 DM dataset	20	X	X		X		Clarke error 80.4% Zone A, 18.1% Zone B
Aiello, Jaloli, and Cescon, 2024 [131]	LSTM-MPC<br><br>ARX-MPC	Stochastic meal generator<br>Scenario-I: Insulin therapy based only on the administration of the basal insulin throughout the day.<br>Scenario-II: Insulin therapy based on the administration of basal insulin together with insulin boluses at mealtimes.	120 minutes	In silico	10 subjects	x		x	x		TIR: 74.99 ± 7.09 vs. 54.15 ± 14.89;<br>TITR: 47.78 ± 8.55 vs. 34.62 ± 9.04;<br>Time in severe hypo: 1.00 ± 3.18 vs. 9.45 ± 11.71

Table S3: Summary of Hybrid Blood Glucose Prediction Model Approaches

Author	Model Type	Additional Aspects	Sub-Systems	Prediction Horizon	Subjects Used		Inputs Used					Accuracy
					Real Data	Simulated Data	CGM / BG	Simple Carbohydrate	Mixed Meals	Insulin	Additional Aspects	
Hutten, 1990 [132]	Black-box model	Multi-compartment physiological model	Glucose–insulin compartment				x		x	x	Gender, age, weight, height, body type, physical activity	
Parker et al., 1999 [133]	Linear step-response model	Kalman filter	Glucose–insulin systems			x		x			Glucose uptake from the gut (Lehmann and Deutsch model)	15mg/dL
Hovorka et al., 2004 [58]	Non-linear model predictive control	Bayesian parameter estimation	Glucose–insulin kinetics; glucose sub-system; insulin sub-system; insulin action sub-system	15–240 minutes	5 x 2		x	x		x		Clarke error grid 95% zone A and 5% zone B (60 minutes); 66% zone A, 33% zone B, 1% zone C, and 0.1% zone E (240 minutes)
Mougiakakou, Prountzo, and Nikita, 2005 [134]	Combination: compartmental models and artificial neural networks	Feed-forward NN; recurrent NN; free-run RNN-FR, and teaching-force RNN-TF	Compartmental model: glucose absorption from the gu/insulin kinetics		275 glucose measurements			x		x		RMSE RTRL-FR 41, RTRL-RF 45
Roy and Parker, 2006 [135]	Model predictive control	Extended minimal model linearized using first-	Bergman minimal model	350 minutes			x		x		Gut absorption of glucose, protein, and fat	

		order Taylor series										
Schlotthauer et al., 2006 [136]	Non-linear model predictive control	Lavenberg – Marquardt algorithm	Physiological patient model				x	x		x	Carbohydrate absorption	
Kildegaard et al., 2007 [137]	AI model	Gaussain-shaped weighting	AIDA - physiological model simulating BG, plasma insulin, and carbohydrate absorption		12		x		x	x		
Markakis et a, 2008 [138]	Model predictive control	Non-parametric / principle dynamic modes model	Augmented minimal model. Glucose regulation (Sorensen's model)				x		x	x		
Ståhl and Johansson, 2009 [139]	ARMAX model	GTFM - Wiener model	Glucose, glucose–insulin, and glucose–insulininteraction sub-model	120 minutes	56 datasets			x		x		95% <1mmol/L
Estrada, Del Re, and Renard,2010 [140]	Linear model with physiologically derived inputs	Normalized least mean squares (NLMS)	Glucose–insulin sub-system, plasma insulin model	45 minutes	15		x		x (meal model)	x		CG-EGA 97.35% clinically accurate
Estrada et al., 2010 [137]	Autoregressive model with exogenous input ARX	Normalized least mean square method with constant and adaptive gain	Glucose–insulin sub-system, mealmodel, plasma insulin, Aspart model, plasma insulin, Determir model	45 minutes	4		x	x		x		92.99% zone A (hypoglycemic) 91.99% zone A (Euglycemic), 96.34% zone A hyperglycemic
Calm et al.,2011 [19]	Monte Carlo simulation (MCS) with modal interval analysis (MIA)		Insulin absorption and appearance rate, intestinal glucose absorption, glucose appearance rate, insulin–glucose sub-system			4	x	x		x	Variation in carbohydrate rate (%), variation insulin sensitivity (%)	
Georga, Protopappas ,	Support vector machines for regression	Physiological processes-related		15/30/60/ 120 minutes			x		x (glucose appearance	x	Exercise	RSME pt 1 - 12.57 (15m), 21.36 (30m),

and Fotiadis, 2011 [141]		compartmental models (s/c insulin, absorption of glucose from the gut, effects of plasma glucose and insulin dynamics)							nce after meal)			33.06 (60m),62.29 (120m). Pt2 - 9.69 (15m), 16.32 (30m), 34.52 (60m), 31.10 (120m). CEGA - Zone A 73.84%, zone B 24.66%
Percival et al.,2011 [142]	Multi-parametric model predictive control	Gaussian white noise, second order plus time delay transfer function	Hovorka model/ Wilinska insulin absorption model	3 hours		10 x 2	x	x		x	Carb-to-insulin ratio, carbohydrate, CF	RMSE 26mg/dL
de Pereda et al., 2012 [143]	Ordinary differential equations		Hovorka glucose–insulinmodel	30 minutes		x		x (digestion and absorption)		x		
Bunescu, 2013 [27]	Modified Lehman and Deutsch glucose absorption model and support vector regression	Meal absorption dynamics; insulin dynamics and glucose dynamics		30–60 minutes	10	x	x		x	Exercise and sleep data	RSME 18mg/dl (30 minutes)and 30.9mg/dl (60 minutes)	
Toffanin et al.,2013 [74]	Model predictive control	Kalman filter	Glucose–insulin state			UVa/Padova		x			Carb-to-insulin ratio and correction factor	
Georga *et al.*,2013 [24]	Multivariant regression using support vector regression	Gaussian radial basis function optimized with differential evolution algorithm, 10-fold	Insulin model (absorption kinetics), meal model (Lehmann and Deutsch)	15/30/60/ 120 minutes		27 pts datasets	x	x		x	Energy expenditure, food diary (portion size, mealtime, etc.)	Average errors 5.21 (15m), 6.03 (30m), 7.14 (60m), and 7.62 (120m)

		cross validation										
Gondhalekar et al., 2013 [144]	Periodic zone model predictive control (PZMPC)		Insulin–glucose model (van Heusden et al.)			100 (in silico UVa/Padova)	x	x (unannounced)		x		
Toffanin *et al.,* 2013	Linear model predictive control (LMPC)	Kalman filter	Insulin–glucose dynamics			47	x	x		x		
Bock, François, and Gillet, 2015 [56]	Stochasti cmodel	Extended Kalman filter; therapy parameter-based model	Gut glucose absorption			10 (Uva/ Padova)		x		x		
Cescon, Johannson, Renard, 2015 [145]	Subspace-based linear multi-step predictors; discrete time-linear time-invariant system	Hankel matrices; Kalman filters	Glucose model/ insulin model/ glucose–insulin interaction (Dalla Man model)	30/60/90/ 120 minutes			x	x		x		
Zecchin et al.,2016 [87]	Feed-forward neural network		Dalla Man absorption model	15/30/45/ 60 minutes	15		x	x		x	Compares CGM vs CGM + Insulin vs CGM + carbohydrate vs CGM + Insulin + carbohydrate	
Contreras et al., 2017 [28]	Hybrid approach	Grammatical Evolution/ physiological modeling (Hovorka)/	01:00 - 06:59/ 07:00 - 12:59/ 13:00 - 18:59/ 19:00 - 00:59	6 hours	20	100 virtual (Uva/Padova)	x	x		x	Glucagon dynamics	98.31% zones A and B (Clarke error grid)/ RMSE: Breakfast (testing) - 24.60mg/dL; Lunch (testing) - 23.20mg/dL; Dinner (testing) - 33.00mg/dL; 24 (testing) - 23.25mg/dL

Mirshekarian et al., 2017 [146]	Recursive neural network (RNN)	Long short-term memory (LSTM)/ support vector regression (SVR)/ extended Kalman filter (EKF)	Meal absorption dynamics; insulin dynamics; glucose compartment; glucose–insulin concentrations	30/60 minutes	5		x	x (mealtime and carbohydrate content)	x	x (bolus - time, type, and amount AND basal rate)		RMSE: vanilla RNN 225 (30 minutes) and 40.5 (60 minutes)/ LSTM 21.4 (30 minutes) and 38.0 (60 minutes)
Reitter, Reitrer, and delRe 2017 [87]	Markov chain model	Bayesian network	Carbohydrate absorption in the gut, absorption, and action of insulin	30/60 minutes	37		x	x		x		
Bertachi et al.,2018 [147]	Regression model (ANN)	Physiological models (insulin on board, CHO on board, and activity on board)	Carb on board (Hovorka model), IOB (Wilinska et al.)				x	x		x	Activity (step count)	RMSE (ave) 19.33 (30m), 31.72 (60m)
Contreras et al., 2018 [148]	Grammatical evolution		Insulin on board model; activity on board model; carb absorption model	30/60/90 minutes			x	x		x	Activity	RSME (average) 21.19 (30 minutes); 31.34 (60 minutes); 36.26 (90 minutes)
Hajizadeh et al., 2018 [149]	Predictor-based subspace identification	Unscented Kalman filter	Insulin sub-system (Hovorka model)	30 minutes	10 pts		x	x		x	Exercise (two sessions 20–30 minutes before and after lunch)	RMSE 22.50mg/dL (30 minutes)
De Falco et al., 2019 [150]	Neuroevolution of augmenting topologies (NEAT)	Padding pre-processing for glucose values, Bergman model for pre-processing of insulin	Gut glucose absorption	30/60 minutes	6 - OhioT1DM dataset		x	x		x		RMSE (mg/dL) 20.636 (average); 20.494 (best)

Hajizadeh et al., 2019 [151]	Model predictive control (MPC)	Recursive predictor-based sub-space identification (PBSID; linear time varying glycemic models	Glucose–insulin dynamics (Hovorka); plasma–insulin risk index; plasma–insulin concentration bounds	20 minutes			x	x		x	Exercise	
Knopp et al., 2019 [152]	Two-compartment gut model	NICING glucose–insulin model for very premature infants	Glucose in the stomach and in the intestine		4 (Budapest) and 10 (SUGAR-BABIES)		x	x				
Dias, Kamath, Vidyasagar, 2020 [153]	Model predictive control (MPC)	Self-organizing map (SoM)	Glucose, insulin, glucagon model in ODE form				x	x		x		4.4mg/dL average tracking error
Goyal et al., 2020 [154]	Discrete event simulator in an object-oriented manner (SIMulation ConTroLler object and HumanBody Object)		Food, exercise; insulin production; glucose transport; gluconeogenesis; glycolysis; liver glycogen synthesis and breakdown				x	x		x	GI, Protein, and fat per serving; exercise	
Karim, Vassányi, and Kósa, 2020 [7]	Feed-forward artificial neural network		Arleth absorption model	60/90/120/180 minutes			x	x		x	Absorption profiles	RMSE 1.49mmol/L (60m); 1.62mmol/L (90m); 1.72mmol/L (120m); 1.95mol/L (180m)
Montaser et al., 2020 [155]	SARIMAX; Fuzzy C-means clustering algorithm	Unscented Kalman filter	Modified Bergman's minimal model	15/30/45/60/90 minutes			x	x		x	Physical activity, multivariant adaptive artificial pancreas system (MAAP)	ED: 5.26 (15 minutes); 7.4 (30 minutes); 9.31 (45 minutes); 11.10 (60 minutes); 12.92(90 minutes)

Pereira et al.,2020 [30]	Hybrid model (ODEs; agent-based models (ABMs))	Runge–Kutta fourth order method	Human glucose–insulin regulation (HGIRS); computational models				x	Carbohydrate and fat		x	Physical activity	
Adelberger et al., 2021 [156]	Kirchsteiger model	Process model combined with Kalman filter (KF-PM); hybrid process and autoregressive model (AR-PM)		45/60/90 minutes	175		x	x		x	Carb-to-insulin ratio and insulin sensitivity factor	ZOH breakfast 4.3 (45m), 25.7 (60m), 25.0 (90m); Lunch 33.3 (45m), 27.3 (60m), 21.4 (90m); Dinner 48.1 (45m), 33.3 (60m), 25.0 (90m)
Cervigón, Velasco, and Burgos-Simón, 2021 [157]	Real-coded genetic algorithm	Data augmented by Gaussian white noise; daily samples divided into 12 equally sized slots; genetic algorithm to adjust minimal model; time series outcome from previous stage adjusted using probabilistic fitting; averaging the individual predictions of the ensemble's model	Prud'homme et al. adapted Bergman minimal model	24 hours	4 (in patient)		x	x		x	Physical activity, time of day	Parker error grid: Pt 1 all results in zones A and B bare one exception; Pt 2mainly zones Aand B high percentage in zone c; Pt 3 most points in zone B small percentage in zone C; Pt 4 high percentage points in zone C

Karim, Vassányi, and Kósa, 2021 [10]	Feed-forward artificial neural network	Quasi-Newton (training)	Meal absorption (Arleth method); AUC whole absorption curve, time lapsed from basal injection	120–180 minutes	5 (1TD, 4 T2D)		x		x	x	Meal absorption, time since basal injection	RSME 1.72mmol/l (120min) and 1.95mmol/L (180 minutes)
Martínez-Delgado, Munoz-Organero, and Quiepo-Alvarez, 2021 [42]	Recurrent neural network (LSTM)	Insulin pre-processing and food data pre-processing	Carbohydrate absorption curves and Haiya et al. insulin absorption model	60 minutes	D1NAMO dataset - 6 T1DM		x	x		x	Glycemic index	RMSE (mmol/L) 0.510 (best case scenario: 1 pt mean)
Muñoz-Organeron et al., 2021 [42]	Recurrent neural network based on LSTM	Unscented Kalman filter, mutGaussain parameter	Horvorka's glucose–insulinmodel	30/60 minutes		40	x	x (meal intake)		x	Physical activity, stress, hormones	Two-layer RNN: average mean error 0.077; single layer RNN: average mean error 0.088
Tavarez et al.,2022 [158]	Stochasticmodel	Kalman filter	First order dynamics are added to physiological model for BG meter, absorption of glucose in gut	1/15 minutes		1	x	x		x		Kalman filter with CHO and EI delivery achieved improvements > 50%
